# Remarks on Limit Theorems for the Free Quadratic Forms

**DOI:** 10.3390/e26100870

**Published:** 2024-10-17

**Authors:** Wiktor Ejsmont, Marek Biernacki, Patrycja Hęćka

**Affiliations:** 1 Department of Telecommunications and Teleinformatics, Wrocław University of Science and Technology, Wybrzeże Wyspiańskiego 27, 50-370 Wrocław, Poland; wiktor.ejsmont@gmail.com; 2 Department of Mathematics and Cybernetics, Wroclaw University of Economics and Business, Komandorska st. 118/120, 53-345 Wrocław, Poland; marek.biernacki@ue.wroc.pl

**Keywords:** limit theorems, tangent law, commutator

## Abstract

In 2021, Ejsmont and Biernacki showed that the free tangent distribution can be used to measure household satisfaction with durable consumer goods. This distribution arises as the limit of free random variables. This, new article serves as the theoretical introduction to the continuation of the research presented in the paper from 2021. We continue the study of the limit of specific quadratic forms in free probability, which is the first step towards constructing a new distribution for the evaluation of satisfaction with material affluence among household. We formulate a non-central limit theorem for weighted sums of commutators and square of the sums for free random variable. In addition we give the random matrix models for these limits.

## 1. Introduction

Voiculescu introduced free probability [1,2] 30 years ago to solve some problems in von Neumann algebras of free groups, giving rise to a whole new field with connections to different branches of mathematics, like, e.g., classical probability, analysis and combinatorics, random matrices [3], operator algebras or noncrossing partitions [4].

This relatively new field is considered to be the most developed branch of noncommutative probability. Many mathematicians have discovered various connections between classical and free world. One of the most important is a central limit theorem with the Wigner semicircle law appearing in the limit. Other famous discoveries include corresponding Brownian motion and the existence of the Bercovici–Pata bijection [5] between infinitely divisible distributions in the classical and the free probability. For example, it turns out that for a given family of free identically distributed random variables $X_{1},\ldots ,X_{n}$ with a mean equal to 0 and variance equal to 1, the distribution of

$$\frac{X_{1}+\cdots +X_{n}}{\sqrt{n}},$$

converges in distribution to the Wigner semicircle law with density

(1) $$d\mu \left(x\right)=\frac{1}{2\pi }\sqrt{4-x^{2}}\;dx$$

on −2≤x≤2, as *n* goes to infinity. It is the free analogue of the central limit theorem. In 2020, Ejsmont and Lehner investigated a limit theorem for weighted sums of free anticommutators and commutators [6], as follows:

$$\begin{array}{c} T_{n}=\frac{1}{n}\sum_{k<l}a\left(X_{k}X_{l}+X_{l}X_{k}\right)+bi\left(X_{k}X_{l}-X_{l}X_{k}\right) \end{array}$$

where a,b∈R, such that $a^{2}+b^{2}=1$ and b≠0 for free random variables. In the present paper, we continue this topic and compute a limit theorem for the sums

$$\begin{array}{cc} \frac{1}{n}\left(\sum_{k,l}aX_{k}X_{l}+ib\sum_{k<l}\left(X_{k}X_{l}-X_{l}X_{k}\right)\right) & =T_{n}+\frac{a}{n}{\left(\sum_{k}X_{k}\right)}^{2}. \end{array}$$

As a result of these studies, we once again obtained the resealing tangent function. We note that this result is not directly obvious because the additional factor ${\left(\sum_{k}X_{k}\right)}^{2}$ is not freely independent with $T_{n}.$ Many mathematicians studied central or non-central limit theorems in classical probability, e.g., [7,8,9]. In the classical world, commutator $X_{k}X_{l}-X_{l}X_{k}$ is not usually considered because it is equal to 0. The reader can also consult the studies about the limit of weighted sums of Boolean commutators and anticommutators [10]. We encourage the reader to read the article showing that the free tangent distribution can be used to measure household satisfaction with durable consumer goods [11].

## 2. Preliminaries

### 2.1. Basic Notation and Terminology

A tracial noncommutative probability space consists of a pair (A,τ), where A is a ∗-algebra, and τ:A→C is a normal, faithful, tracial state. This means that τ is linear, continuous in the weak* topology, satisfies τ(XY)=τ(YX), with τ(I)=1, and $\tau (XX^{*})\geq 0$ and $\tau (XX^{*})=0$, implying that X=0 for all X,Y∈A.

The elements X∈A are referred to as noncommutative random variables. In this paper, all random variables are assumed to be self-adjoint. For a noncommutative random variable $X\in A_{sa}$, the spectral theorem gives a unique probability measure $\mu_{X}$ on R, which represents the distribution of *X* in the state τ. In particular, $\tau \left(f\left(X\right)\right)=\int_{R}f\left(\lambda \right)\;d\mu_{X}\left(\lambda \right)$ for any bounded Borel function *f* on R.

### 2.2. Free Independence

A collection of subalgebras ${\left(A_{i}\right)}_{i\in I}$ of A is said to be *free* if $\tau (X_{1}\ldots X_{n})=0$ whenever $\tau (X_{j})=0$ for all j=1,…,n and $X_{j}\in A_{i(j)}$ with distinct indices i(1)≠i(2)≠…≠i(n). Random variables $X_{1},\ldots ,X_{n}$ are considered freely independent if the subalgebras generated by these variables are free.

### 2.3. Free Convolution and the Cauchy–Stieltjes Transform

It can be proven that the joint distribution of free random variables $X_{i}$ is completely determined by the distributions of the individual $X_{i}$, which leads to a well-defined operation known as *free convolution*: Let μ and ν be probability measures on R, and let X,Y be self-adjoint free random variables with distributions μ and ν, respectively. The distribution of X+Y is referred to as the free additive convolution of μ and ν, which is denoted by μ⊞ν. The analytic approach to free convolution relies on the use of the Cauchy transform

(2) $$\begin{array}{c} G_{\mu }\left(z\right)=\int_{R}\frac{1}{z-y}\;d\mu \left(y\right) \end{array}$$

of a probability measure μ. The Cauchy transform is analytic on the upper half plane $C^{+}=\left\{x+iy|x,y\in R,y>0\right\}$ and maps to the closed lower half plane $C^{-}\cup R$. For measures with compact support, the Cauchy transform is analytic at infinity and connected to the moment generating function $M_{X}$, as follows:

(3) $$\begin{array}{c} M_{X}\left(z\right)=\sum_{n=0}^{\infty }\;\tau \left(X^{n}\right)\;z^{n}=\frac{1}{z}\;G_{X}\left(1/z\right). \end{array}$$

Furthermore, the Cauchy transform has an inverse in a neighborhood of infinity, which can be expressed as

$$G_{\mu }^{-1}\left(z\right)=\frac{1}{z}+R_{\mu }\left(z\right),$$

where $R_{\mu }\left(z\right)$ is analytic near zero and is known as the *R-transform*. The coefficients of its series expansion

(4) $$\begin{array}{c} R_{X}\left(z\right)=\sum_{n=0}^{\infty }\;K_{n+1}\left(X\right)z^{n} \end{array}$$

are called the *free cumulants* of the random variable *X*. The *R*-transform has a key property: it linearizes free convolution. That is, for measures μ and ν,

(5) $$\begin{array}{c} R_{\mu ⊞\nu }\left(z\right)=R_{\mu }\left(z\right)+R_{\nu }\left(z\right). \end{array}$$

### 2.4. Convergence in Distribution

In noncommutative probability, a sequence of random variables $X_{n}$ is said to *converge in distribution* to *X* as n→∞, which is written as

$$X_{n}\overset{d}{\to }X$$

if for every m∈N, the following holds:

$$\lim_{n\to \infty }\tau \left(X_{n}^{m}\right)=\tau \left(X^{m}\right)\;\mathrm{or}\;\mathrm{equivalently}\lim_{n\to \infty }K_{m}\left(X_{n}\right)=K_{m}\left(X\right),$$

where $K_{m}$ denotes the free cumulants of the random variables.

### 2.5. Random Matrices

In this subsection, we remind the reader of the definitions concerning random matrices that are needed to formulate our later results. Consider an N×M *complex Gaussian random matrix* $X={\left[x_{i,j}\right]}_{i,j=1}^{N\times M}$, where each entry $x_{i,j}$ is an independent and identically distributed complex Gaussian random variable with a mean equal to zero and variance of $E(|x_{i,j}{|}^{2})=\frac{1}{N}$. Such matrix is said to be from the Ginibre unitary ensemble. Specifically, the real and imaginary components of $x_{ij}$, denoted by Rexij and Imxij, are independent random variables, which together form an i.i.d. family of $N(0,\frac{1}{2N})$ random variables.

**Remark 1.**  *In the next subsection, we recall the tangent numbers $T_{2k-1}$—see Equation (6). We used a similar symbol in Equation (1). We would like to emphasize that these are two different objects.*

### 2.6. Combinatorics of Tangent Numbers

*Tangent numbers* are given by the following formula:

(6) $$T_{2k-1}={\left(-1\right)}^{k+1}\frac{4^{k}\left(4^{k}-1\right)B_{2k}}{2k}$$

for k∈N, where $B_{2k}$ represents the 2k-th Bernoulli number. Numbers $T_{2k-1}$ are the Taylor coefficients of the tangent function, expressed as

$$\tan z=\sum_{n=1}^{\infty }T_{n}\frac{z^{n}}{n!}=z+\frac{2}{3!}z^{3}+\frac{16}{5!}z^{5}+\frac{272}{7!}z^{7}+\cdots ,$$

as detailed in [12], p. 287. Moreover, tangent numbers are complemented by *secant numbers*. Together, they form the sequence of $E_{n}$, known as the *Euler zigzag numbers*, which represent the Taylor coefficients of the function

$$\tan z+\sec z=\sum_{n=0}^{\infty }\frac{E_{n}}{n!}z^{n}.$$

It is well established that all derivatives of the tangent function can be expressed by specific polynomials, as noted in [12], p. 287. More precisely, there exists a sequence of polynomials $P_{n}\left(x\right)$ of degree n+1, such that

$$\begin{array}{cc} \frac{d^{n}}{d\theta^{n}}\tan\theta & =P_{n}\left(\tan\theta \right). \end{array}$$

The generating function is equal to

(7) $$\tan\left(\theta +z\right)=\sum_{n=0}^{\infty }\frac{P_{n}\left(\tan\theta \right)}{n!}z^{n}=\frac{\tan\theta +\tan z}{1-\tan\theta \tan z}.$$

### 2.7. Complementary Facts

In this subsection, we will present some general limit theorems for quadratic forms, namely [6] (Theorem 3.1) and [6] (Corollary 3.5), which will serve as the main technical tools for the proofs.

**Theorem 1.**  *Let $A_{n}=\left[a_{i,j}^{\left(n\right)}\right]\in M_{n}\left(C\right)$ be a sequence of self-adjoint matrices satisfying the condition that $\sup_{i,j,n}\left|a_{i,j}^{\left(n\right)}\right|<\infty$. Furthermore, the matrix $\frac{1}{n}A_{n}$ converges in distribution to a limiting distribution μ with respect to the trace, meaning that*

$$\lim_{n\to \infty }\mathrm{error}\left({\left(\frac{1}{n}A_{n}\right)}^{r}\right)=\int t^{r}d\mu \left(t\right)$$

*holds for all r∈N. Consider $X_{i}$ as free copies of a centered random variable X with a variance of 1; thus, the sequence of quadratic forms*

$$Q_{n}=\frac{1}{n}\sum_{i,j=1}^{n}a_{i,j}^{\left(n\right)}X_{i}X_{j}$$

*converges in distribution to Y, characterized by*

$$K_{r}\left(Y\right)=\int t^{r}d\mu \left(t\right).$$

For complex scalars a,b,c∈C, we define the matrix ${\left[\begin{array}{cc} c & a\\ b & c \end{array}\right]}_{n}\in M_{n}\left(C\right)$ as one that has *c* on its diagonal, *a* in the upper triangular position, and *b* in the lower triangular position. Then, we can formulate the random matrix model for Theorem 1.

**Theorem 2.**  *Consider the complex Gaussian random matrix $X_{N\times NM}$ and a sequence of self-adjoint M×M matrices $A_{M}=\left[a_{i,j}^{\left(M\right)}\right]$, as outlined in Theorem 1. Let $P_{N}$ be a sequence of deterministic N×N matrices whose moments with respect to the normalized trace converge to 1. Examples include the identity matrix $P_{N}={\left[\begin{array}{cc} 1 & 0\\ 0 & 1 \end{array}\right]}_{N}$ or the projection matrix of large ranks, such as $P_{N}={\left[\begin{array}{cc} 1 & 0\\ 0 & 1 \end{array}\right]}_{N}-\frac{1}{N}{\left[\begin{array}{cc} 1 & 1\\ 1 & 1 \end{array}\right]}_{N}$). Under these conditions, the spectral measures of*

$$\frac{1}{M}X_{N\times NM}\left[A_{M}⊗P_{N}\right]X_{N\times NM}^{*}$$

*will converge in distribution to the limit law, which is presented in Theorem 1.*

## 3. The Main Result

The main contribution of this paper is the following limit theorem, which has a very elegant form. The proof utilizes the techniques presented in [6] and proceeds in a similar manner.

**Theorem 3.**  *Consider free, centered copies of a random variable $X_{1},X_{2},\ldots ,X_{n}\in A_{sa}$ with a variance of 1. Then, for any $\alpha \in (-\frac{\pi }{2},\frac{\pi }{2})$, c∈R, the limit law*

$$Q_{n}=\frac{1}{n}c\left(\tan\alpha \sum_{\begin{array}{c} k,l=1 \end{array}}^{n}X_{k}X_{l}+i\sum_{\begin{array}{c} k<l \end{array}}^{n}\left(X_{k}X_{l}-X_{l}X_{k}\right)\right)\overset{d}{\to }Y,$$

*has an R-transform*

$$R_{Y}\left(z\right)=c\tan\left(cz+\alpha \right).$$

*The free cumulants are given by*

$$K_{r}\left(Y\right)=\frac{c^{r+1}}{r!}P_{r}\left(\tan\alpha \right).$$

**Proof.**  The proof is based on Theorem 1. Let a=ctanα and b=c. The system matrix from Theorem 1 is $\frac{1}{n}A_{n}=\frac{1}{n}{\left[\begin{array}{cc} a & a+ib\\ a-ib & a \end{array}\right]}_{n}$. The characteristic polynomial $\chi_{n}\left(\lambda \right)=\det\left(\lambda I-\frac{1}{n}A_{n}\right)$ satisfies a specific recurrence relation. Let $w=\frac{a}{n}+\frac{ib}{n}$. If we subtract the second row from the first row and the second column from the first column, then we obtain

χn(λ)=λ−an−w−w−w…−w−w¯λ−an−w−w…−w−w¯−w¯λ−an−w…−w−w¯−w¯−w¯λ−an…−w…………………−w¯−w¯−w¯−w¯…λ−an=λ−an+w¯−λ−w+an00…0−w¯λ−an−w−w…−w−w¯−w¯λ−an−w…−w−w¯−w¯−w¯λ−an…−w………………………−w¯−w¯−w¯−w¯…λ−an=2λ+w+w¯−2an−λ−w+an00…0−λ−w¯+anλ−an−w−w…−w0−w¯λ−an−w…−w0−w¯−w¯λ−an…−w…………………………0−w¯−w¯−w¯…λ−an=2λ−λ−ibn00…0−λ+ibnλ−an−w−w…−w0−w¯λ−an−w…−w0−w¯−w¯λ−an…−w……………………0−w¯−w¯−w¯…λ−an=2λχn−1(λ)−λ+ibnλ−ibnχn−2(λ)

and the solution to this recurrence equation, given the initial conditions $\chi_{0}\left(\lambda \right)=1$ and $\chi_{1}\left(\lambda \right)=\lambda -\frac{a}{n}$, which is expressed as

(8) $$\chi_{n}\left(\lambda \right)=\frac{\left(a+ib\right){\left(\lambda -\frac{ib}{n}\right)}^{n}-\left(a-ib\right){\left(\lambda +\frac{ib}{n}\right)}^{n}}{2ib}.$$

Below, we use the result described in detail in [6], which outlines that the cumulant generating function can be obtained from the logarithmic derivative of the characteristic polynomial. Thus, the cumulant generating function

$$R_{Q_{n}}\left(z\right)=\sum_{k=1}^{\infty }\frac{\mathrm{error}(A_{n}^{k})}{n^{k}}z^{k-1},$$

is derived as follows (logarithmic derivative of the characteristic polynomial):

$$\begin{array}{cc} R_{Q_{n}}\left(z\right) & =\frac{1}{z}\left(\frac{1}{z}\frac{\chi_{n}^{\prime }\left(1/z\right)}{\chi_{n}\left(1/z\right)}-n\right)\\ \; & =\frac{n}{z}\left(\frac{w{\left(1-\frac{zib}{n}\right)}^{n-1}-\bar{w}{\left(1+\frac{zib}{n}\right)}^{n-1}}{w{\left(1-\frac{zib}{n}\right)}^{n}-\bar{w}{\left(1+\frac{zib}{n}\right)}^{n}}-1\right)\\ \; & =\frac{ibw{\left(1+\frac{z\overset{¯}{ib}}{n}\right)}^{n-1}+ib\bar{w}{\left(1+\frac{zib}{n}\right)}^{n-1}}{w{\left(1+\frac{z\overset{¯}{ib}}{n}\right)}^{n}-\overset{¯}{w}{\left(1+\frac{zib}{n}\right)}^{n}}, \end{array}$$

where $\chi_{n}^{\prime }\left(1/z\right)={\left(\frac{d\chi_{n}}{dx}\right)}_{x=\frac{1}{z}}$.

Then, the limit is

$$\begin{array}{cc} \lim_{n\to \infty }R_{Q_{n}}\left(z\right) & =R_{Y}\left(z\right)=\frac{ibwe^{z\overset{¯}{ib}}+ib\bar{w}e^{zib}}{we^{z\overset{¯}{ib}}-\overset{¯}{w}e^{zib}},\\ & =\frac{-2bi(b\sin(bz)+acos(bz))}{-2i(a\sin(bz)-b\cos(bz))}=b\frac{a+b\tan(bz)}{b-a\tan(bz)}\\ & =c\frac{\tan\alpha +\tan(cz)}{1-\tan\alpha \tan(cz)}=c\tan\left(cz+\alpha \right). \end{array}$$

Through simple manipulations of formula (7), we obtain the corresponding cumulants. □

In the case where α=0 and b=1, we recover the free tangent law [6].

**Proposition 1**  (Free tangent law [6]). *Consider free copies of a random variable $X_{1},X_{2},\ldots ,X_{n}\in A_{sa}$ with a variance of 1. Then,*

$$Q_{n}=\frac{1}{n}\sum_{\begin{array}{c} k,l=1\\ k<l \end{array}}^{n}i\left(X_{k}X_{l}-X_{l}X_{k}\right)\overset{d}{\to }Y,$$

*where $R_{Y}\left(z\right)=\tan\left(z\right)$.*

The case where $c=\frac{1}{2}$ and $\alpha =\pm \frac{\pi }{4}$ is also interesting because in the limit up to rescaling, we obtain the function tan(z)±sec(z), which appears across various contexts; for instance, it appears in [13,14,15,16], André’s theorem [17], or free zigzag law [6] (Proposition 4.5).

**Proposition 2.**  *Consider free copies of a centered random variable $X_{1},X_{2},\ldots ,X_{n}\in A_{sa}$ with a variance of 1. Then,*

$$Q_{n}=\frac{1}{2n}\left(\pm \sum_{k,l=1}^{n}X_{k}X_{l}+i\sum_{\begin{array}{c} k,l=1\\ k<l \end{array}}^{n}\left(X_{k}X_{l}-X_{l}X_{k}\right)\right)\overset{d}{\to }Y,$$

*where $R_{Y}\left(z\right)=\frac{1}{2}\left(\tan\left(z\right)\pm \sec\left(z\right)\right)$.*

**Proof.**  If we substitute c=1$\alpha =\frac{\pi }{4}$, then by the identity $\tan\left(z\right)\pm \sec\left(z\right)=\tan\left(\frac{z}{2}\pm \frac{\pi }{4}\right)$, we have

$$R_{Y}\left(z\right)=\frac{1}{2}\tan\left(\frac{z}{2}\pm \frac{\pi }{4}\right)=\frac{1}{2}\left(\tan\left(z\right)\pm \sec\left(z\right)\right).$$

□

Finally, as a consequence of Theorem 2, we present a random matrix model for the limit law from Theorem 3.

**Corollary 1.**  *Let $X_{N\times NM}$ be a complex Gaussian random matrix. Then, the spectral measures of*

$$\frac{1}{M}X_{N\times NM}\left[{\left[\begin{array}{cc} a & a+ib\\ a-ib & a \end{array}\right]}_{M\times M}⊗{\left[\begin{array}{cc} 1 & 0\\ 0 & 1 \end{array}\right]}_{N\times N}\right]X_{N\times NM}^{*}$$

*converge in distribution to the limit law outlined in Theorem 3.*

## Data Availability

No new data were created or analyzed in this study. Data sharing is not applicable to this article.
